# Differential Effects of *MYH9* and *APOL1* Risk Variants on *FRMD3* Association with Diabetic ESRD in African Americans

**DOI:** 10.1371/journal.pgen.1002150

**Published:** 2011-06-16

**Authors:** Barry I. Freedman, Carl D. Langefeld, Lingyi Lu, Jasmin Divers, Mary E. Comeau, Jeffrey B. Kopp, Cheryl A. Winkler, George W. Nelson, Randall C. Johnson, Nicholette D. Palmer, Pamela J. Hicks, Meredith A. Bostrom, Jessica N. Cooke, Caitrin W. McDonough, Donald W. Bowden

**Affiliations:** 1 Section on Nephrology, Department of Internal Medicine, Wake Forest School of Medicine, Winston-Salem, North Carolina, United States of America; 2 Department of Biostatistical Sciences and Center for Public Health Genomics, Wake Forest School of Medicine, Winston-Salem, North Carolina, United States of America; 3 Kidney Disease Section, National Institute of Diabetes and Digestive and Kidney Diseases, National Institutes of Health, Bethesda, Maryland, United States of America; 4 Basic Research Laboratory, SAIC-Frederick, National Cancer Institute, National Institutes of Health, Frederick, Maryland, United States of America; 5 BSP CCR Genetics Core, SAIC-Frederick, National Cancer Institute, National Institutes of Health, Frederick, Maryland, United States of America; 6 Chaire de Bioinformatique, Conservatoire National des Arts et Metiers, Paris, France; 7 Department of Biochemistry, Wake Forest School of Medicine, Winston-Salem, North Carolina, United States of America; 8 Center for Diabetes Research, Wake Forest School of Medicine, Winston-Salem, North Carolina, United States of America; 9 Center for Genomics and Personalized Medicine Research, Wake Forest School of Medicine, Winston-Salem, North Carolina, United States of America; 10 Program in Molecular Medicine and Translational Science, Wake Forest School of Medicine, Winston-Salem, North Carolina, United States of America; 11 Section on Endocrinology, Department of Internal Medicine, Wake Forest School of Medicine, Winston-Salem, North Carolina, United States of America; University of Oxford, United Kingdom

## Abstract

Single nucleotide polymorphisms (SNPs) in *MYH9* and *APOL1* on chromosome 22 (c22) are powerfully associated with non-diabetic end-stage renal disease (ESRD) in African Americans (AAs). Many AAs diagnosed with type 2 diabetic nephropathy (T2DN) have non-diabetic kidney disease, potentially masking detection of DN genes. Therefore, genome-wide association analyses were performed using the Affymetrix SNP Array 6.0 in 966 AA with T2DN and 1,032 non-diabetic, non-nephropathy (NDNN) controls, with and without adjustment for c22 nephropathy risk variants. No associations were seen between *FRMD3* SNPs and T2DN before adjusting for c22 variants. However, logistic regression analysis revealed seven *FRMD3* SNPs significantly interacting with *MYH9*—a finding replicated in 640 additional AA T2DN cases and 683 NDNN controls. Contrasting all 1,592 T2DN cases with all 1,671 NDNN controls, *FRMD3* SNPs appeared to interact with the *MYH9* E1 haplotype (*e.g.*, rs942280 interaction p-value = 9.3E^−7^ additive; odds ratio [OR] 0.67). *FRMD3* alleles were associated with increased risk of T2DN only in subjects lacking two *MYH9* E1 risk haplotypes (rs942280 OR = 1.28), not in *MYH9* E1 risk allele homozygotes (rs942280 OR = 0.80; homogeneity p-value = 4.3E^−4^). Effects were weaker stratifying on *APOL1*. *FRMD3* SNPS were associated with T2DN, not type 2 diabetes *per se*, comparing AAs with T2DN to those with diabetes lacking nephropathy. T2DN-associated *FRMD3* SNPs were detectable in AAs only after accounting for *MYH9*, with differential effects for *APOL1*. These analyses reveal a role for *FRMD3* in AA T2DN susceptibility and accounting for c22 nephropathy risk variants can assist in detecting DN susceptibility genes.

## Author Summary

African Americans have high rates of kidney disease attributed to type 2 diabetes mellitus. However, approximately 25% of patients are misclassified and have non-diabetic kidney disease on renal biopsy. The *APOL1*-*MYH9* gene region on chromosome 22 is powerfully associated with non-diabetic kidney diseases in African Americans. Therefore, we tested for interactions between single nucleotide polymorphisms across the genome with *APOL1* and *MYH9* non-diabetic nephropathy risk variants in African Americans with presumed diabetic nephropathy. Markers in *FRMD3*, a gene associated with type 1 diabetic nephropathy in Caucasians, appeared to interact with *MYH9*; however, increased nephropathy risk was seen in diabetic cases lacking two *MYH9* risk haplotypes, and protective effects were seen in those with two *MYH9* risk haplotypes. Stratified analyses based on the chromosome 22 nephropathy risk haplotypes demonstrated that *FRMD3* variants were associated with diabetic nephropathy risk in cases without two *MYH9* (or *APOL1*) risk haplotypes. It appears that African Americans with diabetes and kidney disease who are not chromosome 22 nephropathy risk variant homozygotes are enriched for the presence of diabetic nephropathy and *FRMD3* risk alleles. This genetic dissection ultimately allowed for detection of the *FRMD3* diabetic nephropathy gene association in a subset of cases enriched for this disorder.

## Introduction

Impressive genetic association is observed between single nucleotide polymorphisms (SNPs) on chromosome 22q (c22) and a spectrum of related kidney disorders [1]–[9]. Nearly 40% of end-stage renal disease (ESRD) in African Americans (AAs) may be attributable to c22 nephropathy risk variants, including 70% of non-diabetic ESRD [10]. Fine mapping studies reveal that several independent SNPs and regions in and near the apolipoprotein L1 gene (*APOL1*) and non-muscle myosin heavy chain 9 gene (*MYH9*) are associated with nephropathy susceptibility [11]. The strongest associations are observed with focal segmental glomerulosclerosis (FSGS), Human Immunodeficiency Virus-associated nephropathy (HIVAN or HIV-associated collapsing glomerulopathy), and hypertension-attributed ESRD (HA-ESRD or focal global glomerulosclerosis [FGGS]). Odds ratios (OR) for the *APOL1* G1 (non-synonymous coding variant 342G∶384M) and G2 (6 basepair deletion) range from 10.5 in FSGS to 7.3 in non-diabetic HA-ESRD. In the case of *MYH9*, E1 risk haplotype ORs range from 5–8 in FSGS to 2–3.4 in non-diabetic ESRD. Although the nephropathy association with *MYH9* is markedly attenuated after accounting for the coding variants in *APOL1*, three groups observe independent *MYH9* association with non-diabetic nephropathy (personal communication 2010: Jeffrey Kopp; Carl Langefeld; Linda Kao). Weaker associations were reported between *MYH9* and type 2 diabetes-(T2DM) associated ESRD in AA (OR∼1.4) [4]. One explanation is that a subset of patients thought to have diabetic nephropathy (DN) had FSGS with coincident T2DM. We reported such a case [12] and estimate that this subset approaches 12–16% of AA with clinically diagnosed DN [4].

Approximately 12% of AAs carry two *APOL1* risk variants and are at risk for FSGS and ∼50% with HIV infection will develop HIVAN in the absence of anti-retroviral therapy. Thus, additional modifying environmental and/or inherited factors appear necessary to initiate kidney disease [13], [14]. Since HIV infection increases the risk for nephropathy by a factor of nearly fivefold, it is possible that other environmental or genetic factors interact with *APOL1* and/or *MYH9* to mediate risk of renal disease. Gene-gene interactions are a likely contributor to susceptibility for diabetic and non-diabetic nephropathy and were the focus of these analyses.

A genome-wide association study (GWAS) using the Affymetrix Genome Wide Human 6.0 SNP chip was completed to identify genetic polymorphisms that mediate risk for T2DM-ESRD in AA [15]. Herein, we scanned the genome to detect polymorphisms mediating risk for T2DM-ESRD, conditional on *APOL1* G1/G2 nephropathy risk variants and the *MYH9* E1 risk haplotype using case-control and case-only study designs. The case-only design increases the statistical power over more classic case-control designs, allowing us to maximize power for the first genome-wide scan testing for interactions with the strongest genetic risk factor for ESRD. We tested for replication in additional AA cases with T2DM-ESRD and AA controls with T2DM lacking nephropathy. Together, these study designs have the potential to detect additional genes mediating the risk for T2DM-ESRD in AAs, accounting for the effects of non-diabetic etiologies of nephropathy with evidence for association on c22. We were also able to assess whether the adjacent *APOL1* and *MYH9* genes exhibited similar effects.

## Results

The discovery GWAS association analysis included 952 AA cases with T2DM-ESRD and 988 AA non-diabetic, non-nephropathy controls, as published [15]. Principal component (PC) analysis identified one PC that controlled for global admixture in this sample and yielded an inflation factor of 1.01 (see Table S1 and Figure S1). Replication analyses were performed in 640 additional unrelated T2DM-ESRD cases and 683 non-diabetic, non-nephropathy controls recruited using identical criteria. Finally, an additional 513 AA with T2DM lacking nephropathy were subsequently evaluated to determine whether associations observed between T2DM-ESRD cases and non-diabetic, non-nephropathy controls reflected nephropathy susceptibility or risk of T2DM *per se*. Table 1 displays the numbers in each case and control group that were homozygous for *MYH9* E1 risk haplotypes and had 2 *APOL1* G1 and/or G2 nephropathy risk variants, along with demographic characteristics. Individuals with the *MYH9* E1 haplotype or *APOL1* risk variants (homozygous or heterozygous) tended to have greater estimated West African ancestry based on the principal component analysis (PCA) (p-value<1E^−4^).

**Table 1:** Demographic characteristics of study groups.

Sample (N)	Analysis	MYH9, APOL1 Risk (N)*	Age (years)	Sex (% female)	BMI (kg/m^2^)	Admixture %	Dialysis Duration (years)	T2DM Duration (years)
T2DM-ESRD (952)	Discovery	365, 200	61.65±10.65	61.04	29.68±7.84	0.80±0.11	3.65±4.04	19.80±10.63
Non-T2DM Control (988)	Discovery	308, 110	48.98±11.98	57.17	30.08±7.84	0.78±0.11	N/A	N/A
T2DM-ESRD (640)	Replication	262, 134	60.14±10.16	57.16	missing	0.80±0.11	3.25±3.78	20.72±10.73
Non-T2DM Control (683)	Replication	244, 108	48.54±12.56	51.62	missing	0.77±0.11	N/A	N/A
T2DM Non-Nephropathy (513)	Phenotype Clarification	170, 57	56.84±11.47	65.63	33.42±7.34	0.76±0.12	N/A	10.38±8.99^a^

ACR – urine albumin∶creatinine ratio; N/A not applicable; a – median 8.0; b – median 3.0; c – median 0.9;

*denotes number with two MYH9 E1 risk haplotypes or two APOL1 G1 and/or G2 nephropathy risk variants.

Examination of the top 100 SNP interactions with c22 risk variants (Table S2) identified SNPs within two previously reported nephropathy susceptibility genes, *FRMD3* and *SHROOM3* [16], [17]. These genes were further evaluated. Results of the case-only analysis in the T2DM-ESRD discovery samples revealed that 7 SNPs in *FRMD3* appeared to interact with the *MYH9* E1 haplotype (Table 2), as did 2 SNPs in *SHROOM3*, rs1493360 and rs17002201 (data not shown). *SHROOM3* SNPs failed replication and were not further investigated. The *FRMD3* effects were in the same direction in case-only, case-control and *MYH9* E1 haplotype stratified case-control analyses. These SNPs in *FRMD3* were all in high linkage disequilibrium (LD; Yoruban r^2^ = 0.95–1.0; CEU r^2^ = 1.0) and appeared to confer protective effects against T2DM-ESRD in *MYH9*-E1 risk homozygotes, despite having significant risk effects in non-E1 homozygotes. This effect was less pronounced for *APOL1*. Importantly, there was no evidence of association of *FRMD3* or any of the other top 100 SNPs with T2DM-ESRD in the original GWAS, prior to accounting for these c22 nephropathy risk variants [15].

**Table 2:** Discovery sample chromosome 22-*FRMD3* interaction analysis.

		T2DM-ESRDCase Only Analysis		NDNN ControlOnlyAnalysis		Cases vs.Controls	c22 risk homozygotes	Non-c22 risk homozygotes	c22 risk homozygotes vs.non-risk	
Marker	Gene Interaction	P-ValueAdditive	OR (95% CI)	P-ValueAdditive	OR (95% CI)	HomogeneityP-Value	OR (95% CI)	OR (95% CI)	HomogeneityP-Value*	Classic LogitP-Value**
rs2378658	*MYH9*	6.4E-4	0.70 (0.57,0.86)	6.3E-1	1.06 (0.84, 1.33)	7.4E-3	0.81 (0.61,1.07)	1.27 (1.03,1.56)	1.2E-2	1.4E-2
	*APOL1*	4.3E-2	0.77 (0.6,0.99)	4.0E-1	1.14 (0.84,1.54)	2.1E-2	0.68 (0.45,1.04)	1.13 (0.94,1.37)	3.1E-2	2.9E-2
rs1535753	*MYH9*	5.6E-4	0.69 (0.56,0.85)	5.9E-1	1.07 (0.85, 1.34)	5.9E-3	0.79 (0.60,1.05)	1.27 (1.03,1.57)	8.1E-3	9.5E-3
	*APOL1*	5.4E-2	0.79 (0.61,1.0)	3.3E-1	1.16 (0.86,1.56)	1.9E-2	0.67 (0.44,1.01)	1.13 (0.94,1.37)	2.4E-2	2.2E-2
rs1535752	*MYH9*	4.6E-4	0.69 (0.56,0.85)	6.3E-1	1.06 (0.84, 1.33)	6.1E-3	0.80 (0.6,1.06)	1.28 (1.04,1.57)	9.2E-3	1.1E-2
	*APOL1*	Not tested	-	-	-					
rs942283	*MYH9*	2.5E-4	0.68 (0.55,0.83)	5.5E-1	1.07 (0.85, 1.35)	3.3E-3	0.77 (0.58,1.03)	1.28 (1.04,1.58)	4.7E-3	5.5E-3
	*APOL1*	7.1E-2	0.8 (0.62,1.02)	2.6E-1	1.19 (0.88,1.6)	1.5E-2	0.66 (0.43,0.99)	1.12 (0.93,1.36)	2.1E-2	1.9E-2
rs942280	*MYH9*	1.5E-4	0.67 (0.54,0.82)	4.2E-1	1.1 (0.87, 1.38)	1.5E-3	0.75 (0.57,1.00)	1.34 (1.09,1.65)	1.2E-3	1.4E-3
	*APOL1*	2.2E-2	0.75 (0.59,0.96)	2.6E-1	1.19 (0.88,1.6)	5.3E-3	0.60 (0.4,0.92)	1.17 (0.97,1.41)	5.0E-3	4.1E-3
rs942278	*MYH9*	2.6E-4	0.68 (0.55,0.84)	3.7E-1	1.11 (0.88, 1.4)	1.6E-3	0.80 (0.60,1.06)	1.35 (1.09,1.66)	3.1E-3	3.7E-3
	*APOL1*	7.0E-2	0.8 (0.62,1.02)	2.8E-1	1.18 (0.87,1.59)	8.9E-3	0.67 (0.45,1.02)	1.18 (0.97,1.42)	1.7E-2	1.58E-2
rs10867977	*MYH9*	1.4E-4	0.67 (0.54,0.82)	7.1E-1	1.04 (0.83, 1.32)	4.1E-3	0.85 (0.64,1.12)	1.32 (1.07,1.63)	1.4E-2	1.7E-2
	*APOL1*	Not tested	-	-	-					

952 T2DM-ESRD cases versus 988 non-T2DM, non-nephropathy (NDNN) controls (* Homogeneity P-Value refers to the P-Value from the test for homogeneity of the odds ratio; ** Two-locus logistic regression interaction analysis).

Table 3 contains the replication analysis results in T2DM-ESRD cases and non-diabetic, non-nephropathy controls with 5 of the 7 *FRMD3* SNPs that could easily be multiplexed. The apparent interactive relationship between *MYH9* and *FRMD3* SNPs was maintained despite the smaller sample. Weaker effects persisted for *APOL1*. A combined analysis was then performed using all 1,592 T2DM-ESRD discovery and replication cases relative to all 1,671 non-diabetic, non-nephropathy controls (Table 4). Analyses in T2DM-ESRD cases suggested significant interactions between *FRMD3* SNPs and *MYH9* (*e.g.*, rs942280, p = 9.28E^−7^ additive; OR 0.67, 95% CI 0.57–0.78). Subsequent analyses revealed that *FRMD3* SNP rs942280 (and others) were significantly associated with increased risk for T2DM-ESRD in non-*MYH9* E1 risk haplotype homozygotes (rs942280 OR 1.28, 95% CI 1.09–1.51), but not in *MYH9* E1 risk allele homozygotes (homozygosity p-value comparing the effect of *FRMD3* SNPs in *MYH9* E1 non-risk homozygotes vs. *MYH9* E1 risk homozygotes = 4.82E^−4^). Therefore, the major effect of risk from *FRMD3* on T2DM-ESRD susceptibility was present in non-*MYH9* E1 haplotype homozygotes. Although the direction of effect was the same when replacing the *MYH9* E1 haplotype with *APOL1* risk variants, results were less significant. This could have resulted from the smaller number of *APOL1* risk homozygotes.

**Table 3:** Replication sample chromosome 22-*FRMD3* interaction analysis.

		T2DM-ESRDCases Only		NDNN ControlsOnly		Cases vs.Controls	c22 riskhomozygotes	non-c22 riskhomozygotes	c22 risk homozygotes vs.non-risk	
Marker	GeneInteraction	P-ValueAdditive	OR (95% CI)	P-ValueAdditive	OR (95% CI)	HomogeneityP-Value	OR (95% CI)	OR (95% CI)	HomogeneityP-Value*	Classic LogitP-Value**
rs2378658	*MYH9*	3.7E-3	0.68 (0.52,0.88)	6.8E-1	0.95 (0.73,1.23)	1.5E-1	0.87 (0.63,1.21)	1.23 (0.96,1.58)	9.7E-2	1.0E-1
	*APOL1*	3.6E-2	0.70 (0.50,0.98)	5.2E-1	1.12 (0.79,1.58)	5.1E-2	0.79 (0.51,1.24)	1.14 (0.91,1.43)	1.6E-1	3.4E-1
rs1535753	*MYH9*	7.7E-3	0.70 (0.54,0.91)	7.8E-1	0.96 (0.74,1.25)	1.7E-1	0.84 (0.61,1.17)	1.20 (0.93,1.55)	8.6E-2	8.7E-2
	*APOL1*	4.0E-2	0.71 (0.51,0.98)	4.7E-1	1.14 (0.80,1.61)	4.8E-2	0.77 (0.49,1.21)	1.12 (0.89,1.40)	1.5E-1	2.7E-1
rs942283	*MYH9*	2.1E-3	0.66 (0.51,0.86)	6.2E-1	0.94 (0.72,1.21)	1.4E-1	0.85 (0.61,1.18)	1.22 (0.95,1.58)	8.0E-2	8.2E-2
	*APOL1*	1.9E-2	0.67 (0.48,0.94)	6.6E-1	1.08 (0.76,1.53)	4.8E-2	0.77 (0.49,1.2)	1.12 (0.90,1.41)	1.4E-1	3.3E-1
rs942280	*MYH9*	1.4E-3	0.65 (0.50,0.85)	5.2E-1	0.92 (0.71,1.19)	1.6E-1	0.87 (0.63,1.21)	1.22 (0.95,1.57)	1.1E-1	1.1E-1
	*APOL1*	3.3E-2	0.70 (0.50,0.97)	6.2E-1	1.09 (0.77,1.54)	6.0E-2	0.82 (0.53,1.27)	1.13 (0.90,1.41)	2.1E-1	3.4E-1
rs942278	*MYH9*	1.5E-2	0.72 (0.56,0.94)	5.5E-1	0.92 (0.71,1.20)	3.2E-1	0.94 (0.68,1.30)	1.26 (0.98,1.62)	1.6E-1	1.7E-1
	*APOL1*	5.8E-2	0.73 (0.52,1.01)	6.3E-1	1.09 (0.77,1.54)	9.0E-2	0.86 (0.55,1.35)	1.18 (0.94,1.48)	2.4E-1	4.0E-1

640 T2DM-ESRD cases versus 683 non-T2DM, non-nephropathy (NDNN) controls (* Homogeneity P-Value refers to the P-Value from the test for homogeneity of the odds ratio; ** Two-locus logistic regression interaction analysis).

**Table 4:** Combined chromosome 22-*FRMD3* interaction analysis.

		T2DM-ESRDCases Only		NDNN ControlsOnly		Cases vs.Controls	c22-riskhomozygotes	non-c22 riskhomozygotes	c22 risk homozygotesvs. non-risk	
Marker	GeneInteraction	P-ValueAdditive	OR (95% CI)	P-ValueAdditive	OR (95% CI)	HomogeneityP-Value	OR (95% CI)	OR (95% CI)	HomogeneityP-Value*	Classic LogitP-Value**
rs2378658	*MYH9*	9.5E-6	0.69 (0.59,0.82)	9.2E-1	1.01 (0.85,1.20)	5.7E-3	0.83 (0.67,1.03)	1.25 (1.06,1.46)	2.9E-3	4.0E-3
	*APOL1*	4.5E-3	0.75 (0.61,0.91)	1.9E-1	1.17 (0.93,1.49)	3.6E-3	0.74 (0.55,1.0)	1.13 (0.98,1.31)	1.3E-2	2.6E-2
rs1535753	*MYH9*	1.7E-5	0.70 (0.60,0.82)	8.2E-1	1.02 (0.86,1.21)	5.3E-3	0.81 (0.66,1.01)	1.24 (1.06,1.46)	1.8E-3	2.4E-3
	*APOL1*	6.0E-3	0.76 (0.62,0.92)	1.4E-1	1.20 (0.94,1.52)	3.1E-3	0.72 (0.53,0.98)	1.12 (0.97,1.29)	1.0E-2	1.6E-2
rs1535752	*MYH9*	5.0E-4	0.69 (0.56,0.85)	6.1E-1	1.06 (0.84,1.34)	1.2E-2	0.80 (0.61,1.07)	1.27 (1.03,1.57)	1.1E-2	1.3E-2
rs942283	*MYH9*	2.3E-6	0.68 (0.57,0.79)	8.7E-1	1.01 (0.85,1.20)	2.8E-3	0.80 (0.65,0.99)	1.25 (1.07,1.47)	1.0E-3	1.4E-3
	*APOL1*	3.6E-3	0.74 (0.61,0.91)	1.7E-1	1.18 (0.93,1.5)	2.9E-3	0.72 (0.53,0.97)	1.12 (0.97,1.29)	9.8E-3	1.9E-2
rs942280	*MYH9*	9.3E-7	0.67 (0.57,0.78)	8.1E-1	1.02 (0.86,1.21)	1.6E-3	0.80 (0.65,0.99)	1.28 (1.09,1.51)	4.8E-4	7.0E-4
	*APOL1*	1.2E-3	0.72 (0.59,0.88)	1.6E-1	1.18 (0.93,1.5)	1.3E-3	0.71 (0.52,0.95)	1.15 (0.99,1.33)	4.4E-3	6.7E-3
rs942278	*MYH9*	1.5E-5	0.70 (0.59,0.82)	7.7E-1	1.03 (0.86,1.22)	4.2E-3	0.85 (0.69,1.06)	1.30 (1.11,1.53)	1.6E-3	2.3E-3
	*APOL1*	6.5E-3	0.76 (0.62,0.93)	1.3E-1	1.20 (0.95,1.52)	3.0E-3	0.76 (0.56,1.03)	1.17 (1.01,1.35)	1.2E-2	1.9E-2
rs10867977	*MYH9*	1.7E-4	0.67 (0.54,0.83)	7.8E-1	1.03 (0.82,1.30)	1.2E-2	0.85 (0.64,1.13)	1.31 (1.06,1.62)	1.6E-2	2.2E-2

1,592 T2DM-ESRD Discovery and Replication cases versus 1,671 Discovery and Replication non-diabetic non-nephropathy (NDNN) controls (* Homogeneity P-Value refers to the P-Value from the test for homogeneity of the odds ratio; ** Two-locus logistic regression interaction analysis).

To determine whether the *FRMD3* SNPs were associated with susceptibility to T2DM-ESRD or diabetes *per se*, a final analysis was performed. *FRMD3* allele frequencies were compared between the 513 unrelated AAs with T2DM lacking nephropathy and the 1,592 T2DM-ESRD cases (Table 5). This revealed significant differences in 3 SNP frequencies comparing T2DM-ESRD non-E1 haplotype homozygote cases to controls with T2DM lacking nephropathy (single SNP OR in non-E1 homozygotes and p-value: rs942278 OR 1.26 p = 0.0241; rs942280 OR 1.22 p = 4.73E^−2^; rs1535753 OR 1.21 p = 5.48E^−2^; rs942283 OR 1.18 p = 9.58E^−2^; rs23786558 OR 1.18 p = 9.66E^−2^). These differences were not detectable in non-*MYH9* E1 stratified analyses (data not shown). In addition, *FRMD3* allele frequencies in the 513 AA with T2DM lacking nephropathy were not significantly different compared to the 1,671 non-diabetic, non-nephropathy controls, whether or not stratified on *MYH9* E1 haplotype homozygosity (all p-values>6E^−1^). In addition, significant interactions for *MYH9* were not observed with these 5 *FRMD3* SNPs in an independent NIH series of 283 biopsy-proven FSGS and HIVAN cases vs. 222 non-nephropathy controls, suggesting the effects are limited to T2DM-ESRD (data not shown). Tables S3 and S4 contain *FRMD3* allele frequencies, based on numbers of *MYH9* E1 or *APOL1* risk haplotypes, respectively.

**Table 5:** Chromosome 22-*FRMD3* interaction analysis.

		T2DM-ESRDCases Only		T2DM Non-Nephropathy Controls Only		Cases vs.Controls	c22 riskhomozygotes	Non-c22Homozygotes	c22 risk homozygotes vs.non-risk	
Marker	GeneInteraction	P-ValueAdditive	OR (95% CI)	P-ValueAdditive	OR (95% CI)	HomogeneityP-Value	OR (95% CI)	OR (95% CI)	HomogeneityP-Value*	Classic Logit P-Value**
rs2378658	*MYH9*	9.5E-6	0.69 (0.59,0.82)	7.0E-1	0.94 (0.71,1.26)	5.6E-2	0.80 (0.61,1.05)	1.18 (0.97,1.44)	2.2E-2	2.5E-2
	*APOL1*	4.5E-3	0.75 (0.61,0.91)	5.2E-1	1.12 (0.79,1.58)	3.8E-2	0.62 (0.38,1.01)	1.08 (0.88,1.32)	4.0E-2	1.5E-2
rs1535753	*MYH9*	1.7E-5	0.7 (0.60,0.82)	8.4E-1	0.97 (0.73,1.29)	4.4E-2	0.80 (0.61,1.04)	1.21 (1.00,1.48)	1.3E-2	1.4E-2
	*APOL1*	6.0E-3	0.76 (0.62,0.92)	4.7E-1	1.14 (0.80,1.61)	2.3E-2	0.58 (0.35,0.96)	1.09 (0.89,1.33)	2.2E-2	9.9E-03
rs942283	*MYH9*	2.3E-6	0.68 (0.57,0.79)	5.9E-1	0.92 (0.69,1.23)	5.4E-2	0.78 (0.60,1.03)	1.18 (0.97,1.44)	1.6E-2	1.7E-2
	*APOL1*	3.6E-3	0.74 (0.61,0.91)	6.6E-1	1.08 (0.76,1.53)	2.8E-2	0.58 (0.35,0.95)	1.08 (0.88,1.32)	2.4E-2	1.1E-2
rs942280	*MYH9*	9.3E-7	0.67 (0.57,0.78)	5.3E-1	0.91 (0.68,1.22)	5.1E-2	0.83 (0.63,1.08)	1.22 (1.00,1.49)	2.0E-2	2.3E-2
	*APOL1*	1.2E-3	0.72 (0.59,0.88)	6.2E-1	1.09 (0.77,1.54)	1.5E-2	0.54 (0.33,0.91)	1.14 (0.93,1.39)	8.0E-3	3.4E-3
rs942278	*MYH9*	1.5E-5	0.7 (0.59,0.82)	9.6E-1	1.01 (0.75,1.34)	2.5E-2	0.82 (0.63,1.07)	1.26 (1.03,1.53)	1.1E-2	1.3E-2
	*APOL1*	6.5E-3	0.76 (0.62,0.93)	6.3E-1	1.09 (0.77,1.54)	2.6E-2	0.60 (0.37,1.0)	1.15 (0.94,1.4)	1.8E-2	8.7E-3

1,592 Discovery and Replication T2DM-ESRD cases versus 513 T2DM non-nephropathy controls (* Homogeneity P-Value refers to the P-Value from the test for homogeneity of the odds ratio; ** Two-locus logistic regression interaction analysis).

## Discussion

Herein we report association analysis results accounting for the effects of *APOL1* and *MYH9* on risk of DN in AAs. Stratified and interaction analyses performed in cases with T2DM-ESRD provided an unbiased assessment of potential interactions between both the *APOL1* G1/G2 risk variants and *MYH9* E1 risk haplotype with nearly one million SNPs across the genome. *MYH9* and *APOL1* are strongly associated with non-diabetic ESRD in AAs and can potentially limit ability to detect other nephropathy susceptibility genes with weaker effect. We identified *FRMD3* as potentially interacting with the *MYH9* E1 haplotype in DN susceptibility, an effect replicated in additional AAs with T2DM-ESRD. Our analyses in 3263 AA T2DM-ESRD cases and non-diabetic, non-nephropathy controls revealed an approximate 25–30% increase in DN risk with multiple *FRMD3* SNPs in subjects not homozygous for the *MYH9* E1 risk haplotype (or *APOL1* risk variants). Interaction analyses between *FRMD3* SNPs were repeated in non-diabetic nephropathy cases (with biopsy-proven FSGS and HIVAN) and interactions were not observed. This suggests that the *FRMD3* association is limited to DN. In retrospect, the case-only interaction analyses based on *MYH9* (and *APOL1*) risk variants likely segregated clinically diagnosed cases of T2DM-ESRD into those enriched for non-diabetic nephropathy (c22 nephropathy risk homozygotes with disease in the FSGS spectrum) and non-c22 homozygotes enriched for true DN. The discrepant effect of *FRMD3* protection in c22 nephropathy homozygotes, versus risk in non-c22 nephropathy homozygotes, raised the possibility that the T2DM-ESRD case group contained subsets of cases with different diseases. We suggest that these groups were not comparable based on c22 status [12]. This partitioning allowed for detection of DN association with *FRMD3* SNPs, limited to the non-*MYH9* E1 homozygotes. The analyses were repeated with other SNPs in the complex and extended LD region about the *MYH9* E1 haplotype on c22, including *APOL1*, with comparable directions of results and less significant p-values.

Genetic heterogeneity and gene-gene or gene-environment interactions are frequently hypothesized as being important in complex genetic disorders such as nephropathy. It remains uncommon to formally test and replicate interaction and multiple loci models. We posit that some variant's risk may depend on the influences of other genes or non-genetic factors. Clearly, variants with strong effects such as *MYH9* and *APOL1* are important in and of themselves. However, an important lesson from the current study is that since the *MYH9* E1 haplotype is extremely common in AAs and has a large odds ratio, the E1 haplotype may mask the effects at other loci unless methods are used to account for its influence (*e.g.*, multilocus models, interaction analyses, stratification analyses). We have no reason to expect different results in European-derived populations since *MYH9* risk variants are also strongly associated with non-diabetic ESRD in Europeans and European Americans; however, larger sample sizes would need to be tested due to the markedly lower frequencies of *APOL1* and *MYH9* risk variants.

The challenge of developing effective genetic screening tests is the balance between correctly identifying those variants that correctly and accurately predict individuals who will develop disease and those who will not (i.e., balance between sensitivity and specificity). Here, the *MYH9* E1 haplotype was both an important predictor and clarifier of the contribution of other loci to the risk of nephropathy. Therefore, the search for additional nephropathy susceptibility loci in AAs, conditional on other important loci (*MYH9* and *APOL1*) remains critical. It initially appeared that variants in *FRMD3* were protective and modified risk for developing T2DM-ESRD in AAs with two *MYH9* E1 risk haplotypes; however, these same variants were associated with risk for T2DM-ESRD in non-E1 haplotype homozygotes. This observation suggested that the subset of ESRD cases homozygous for the E1 haplotype differed from non-E1 homozygotes as to their etiology of ESRD and is supported by the observation that biopsy-proven FSGS can be present in AA with T2DM and heavy proteinuria in individuals homozygous for the *MYH9* E1 risk haplotype [12]. Coding variants in *APOL1* are major susceptibility loci for non-diabetic nephropathy; however, independent, weaker *MYH9* effects remain plausible. This report demonstrates that the *FRMD3* association with DN was more readily detectable in non-*MYH9* risk homozygotes, relative to non-*APOL1* risk homozygotes, an observation that supports a potential independent role for *MYH9* in nephropathy susceptibility.

The strong association observed between variants in *APOL1* and near the *MYH9* gene with several kidney diseases was a major breakthrough in our understanding of nephropathy susceptibility in AA [13], [18]. Additional nephropathy susceptibility genes have been identified using GWAS [16], [17], [19]. For example, the 4.1 protein ezrin, radixin, moesin [FERM] domain containing 3 locus (*FRMD3*) was implicated in kidney disease attributed to type 1 diabetes (T1DM) in European Americans from the Genetics of Kidneys in Diabetes (GoKinD) collection, with replication based upon nephropathy progression rates in subjects with T1DM in the Diabetes Control and Complications Trial (DCCT)/Epidemiology of Diabetes Interventions and Complications (EDIC) Study [17]. Despite replication, the GoKinD GWAS failed to reach genome-wide significant evidence of association. It was also unclear whether *FRMD3* was a susceptibility gene for only T1DM-associated nephropathy or contributed to other etiologies of kidney disease. *FRMD3* variants now appear to impact susceptibility to nephropathy from T1DM and T2DM with effects in European- and African-derived populations, apparently not in Japanese [20].

*FRMD3* is expressed in human kidney [17]. The expression profile of *FRMD3* includes human renal mesangial and proximal tubular cells, but has not yet been tested in podocytes [17], [21]. FERM domains are present in a variety of mammalian proteins and the functions of FERM domain-containing proteins, although not completely known, imply that these domains link the plasma membrane with cytoskeletal structures at specific cellular locations by directly binding partner proteins and/or phosphoinositides [22]. *FRMD3* encodes a protein with unknown function [23]; although other 4.1 protein family members are important in maintenance of cell shape [24] and may maintain cell integrity by interacting with transmembrane proteins and actin filaments [25], [26]. Human FERM domain-containing proteins include kinases (focal adhesion kinase [FAK] and Janus kinases [JAKs]), myosins (MYO7, MYO10 and MYO15), phosphatases (protein-tyrosine phosphatase 1E [PTPE1]), ERMs, kindlins, talins, and other less well-characterized proteins. Although FERM-domain containing proteins interact with myosins, we do not feel that our genetic results support *FRMD3* and *MYH9* variants directly interacting to initiate diabetic nephropathy in African Americans. Instead, cases with clinically-diagnosed T2DM-ESRD possessing two copies of the *MYH9* E1 risk haplotype more likely had non-diabetic forms of ESRD (in the FSGS family and mis-diagnosed as T2DM-ESRD). When limiting analyses to non-*MYH9* risk homozygotes, thereby enriching for T2DM-ESRD, the *FRMD3* genetic association became evident. The *FRMD3* SNPs that were associated with T1DM-associated nephropathy in GoKinD samples were subsequently tested in our AA T2DM-ESRD cases and non-diabetic, non-nephropathy controls. These SNPs are not in LD with the 7 SNPs identified in this report (r^2^ = 0.01–0.03 in both Yorubans and CEU). No evidence of association of the GoKinD associated *FRMD3* SNPs was seen in our AA sample with T2DM-ESRD (data not shown).

A limitation of this report was use of a non-diabetic non-nephropathy control group with younger ages and absence of detailed renal phenotyping, relative to T2DM-ESRD cases. Although occult nephropathy in controls would reduce power to detect association, survival-bias could result. Replication in the T2DM non-nephropathy controls likely reduced (but does not eliminate) the potential for survival bias. Similarly, it is difficult to recruit large numbers of AA controls with longstanding T2DM who lack kidney disease, due to the high prevalence of subclinical nephropathy in AAs with diabetes mellitus.

We conclude that variants in *FRMD3* contribute to the risk for nephropathy in AA with T2DM, an effect that was observed only after accounting for *MYH9* (and less so *APOL1*) gene variants and evaluating a subset of AA cases likely enriched for T2DM-associated nephropathy. These analyses replicate the *FRMD3* association in susceptibility to DN, as well as implicate this gene in African-derived populations. In addition to the nephropathy risk imparted by *APOL1* G1 and G2 variants, our results support residual nephropathy risk residing within *MYH9*, or other c22 variants in linkage disequilibrium with the *MYH9* E1 haplotype.

## Materials and Methods

### Patient populations

Diagnostic criteria for T2DM-associated ESRD in Wake Forest University School of Medicine (WFUSM) participants (both discovery and replication samples) includes diabetes diagnosis at age >30 years (in the absence of diabetic ketoacidosis); with either renal histologic evidence of DN or diabetes duration ≥5 years before initiation of renal replacement therapy in the presence of diabetic retinopathy or proteinuria ≥500 mg/24 h and absence of other known causes of nephropathy [3], [15]. Non-diabetic, non-nephropathy controls were recruited to be at low risk for nephropathy based upon the lack of a personal or family history of kidney disease; therefore, renal function testing is not routinely performed due to the low yield of nephropathy. In a subset of 200 non-diabetic, non-nephropathy controls, 98% (196/200) had serum creatinine concentrations <1.5 mg/dl (maximum 1.85 mg/dl). We note that occult kidney disease in non-diabetic, non-nephropathy controls would bias against association and deflate significance. T2DM non-nephropathy controls met criteria for diabetes and had an estimated glomerular filtration rate >60 ml/min and spot albumin∶creatinine ratio <100 mg/g. Among T2DM non-nephropathy controls, 67.5% had diabetes durations exceeding 5 years and 29.6% reported diabetic retinopathy, 57.5% denied retinopathy; and 12.9% were unsure. All subjects provided written informed consent and studies were approved by the WFUSM Institutional Review Board and adhere to the tenets of the Declaration of Helsinki. Clinical criteria for National Institute of Health (NIH) biopsy-proven FSGS cases (229 with idiopathic FSGS; 54 with HIVAN collapsing glomerulopathy) and 222 controls have also been reported [1].

### Genotyping and quality control

Genotyping of the Affymetrix Genome-Wide Human SNP Array 6.0 in the discovery sample of 966 AA cases with T2DM-ESRD and 1032 non-diabetic, non-nephropathy controls was completed at the Center for Inherited Disease Research (CIDR; www.cidr.jhmi.edu) using DNA extracted from peripheral blood. DNA from cases and controls were approximately balanced on each 96-well master plate. A fingerprinting set of 96 SNPs was independently genotyped in all samples and results compared to the corresponding SNPs on the Affymetrix array to confirm sample identity. Genotypes were called using Birdseed version 2; APT 1.10.0 by grouping samples by DNA plate to determine the genotype cluster boundaries. The minimum SNP call rate for an individual was 98.4%. Forty-six blind duplicates were genotyped and had a concordance rate of 99.59%. Cryptic relatedness was identified by the estimated identity-by-descent (IBD) statistics as implemented in PLINK (http://pngu.mgh.harvard.edu/purcell/plink/). There were two unexpected duplicate pairs and 54 unexpected first-degree relative pairs. One of each of these pairs was removed by the following rules: 1) retain T2DM-ESRD cases over non-diabetic, non-nephropathy controls, and 2) if case/control status was congruent, retain the individual with the most complete phenotype data. One individual had a self-reported gender inconsistent with X chromosome genotype data and one had an inbreeding coefficient, F-statistic, more than 4 standard deviations from the mean, both were excluded. The results are based on the remaining 952 T2DM-ESRD cases and 988 non-diabetic, non-nephropathy controls. Replication samples were recruited under identical ascertainment criteria to the discovery samples. *FRMD3* SNPs were genotyped using the iPLEX™ Sequenom MassARRAY platform for replication. Genotyping efficiency >95% and 45 blind duplicates were included to ensure genotyping accuracy. Genotyping FSGS and HIVAN cases and controls were by TaqMan assays available from ABI Biosystems (Foster City, CA).

### Statistical analysis

Each SNP was tested for departure from Hardy-Weinberg Equilibrium (HWE) expectations using a chi square goodness-of-fit test. The primary inference for this conditional/interaction GWAS was the SNPs with <5% missing and no differential missingness between cases and controls, HWE p-value>1E^−4^ in cases and >1E^−2^ in controls and minor allele frequency (MAF) in the entire sample >0.05. A total of 832,357 SNPs met these criteria. However, SNPs that did not meet these criteria were secondarily examined for association with consideration given to potential corroborating evidence of association at flanking SNPs, especially those SNPs with some evidence of HWE departure. The average sample call rate was 99.16% for all autosomal SNPs.

A principal components analysis (PCA) was computed on the 832,357 SNPs to estimate the primary sources of genetic variations, including potential admixture. One principal component (PC) was retained and it correlated highly (r^2^ = 0.87) with previously computed admixture estimates based on 70 ancestry informative markers (AIMs) using the program FRAPPE [27]. The same set of AIMs was genotyped in the replication sample and admixture estimates were computed using FRAPPE. As described below, the GWAS association analyses adjust for the first PC and the replication study and combined analyses adjusted for admixture estimates.

Since not all individuals homozygous for *APOL1* risk variants and/or the *MYH9* E1 risk haplotype develop nephropathy, the probability of developing ESRD may depend on non-genetic factors and other genetic factors interacting with the known c22 risk variants. Thus, a series of complementary logistic regression analyses were computed using the program SNPGWA (www.phs.wfubmc.edu). The analyses were restricted to SNPs with minor allele frequencies >0.10. The primary inference for the following analyses used the additive genetic model for the SNP, provided there was no evidence of departure from the additive genetic model (additive model lack-of-fit test p-value>5E^−2^). If the lack-of-fit to an additive model was significant, then the minimum of the dominant, additive and recessive model is reported. In addition, additive genetic models required at least ten individuals homozygous for the minor allele and recessive models required at least 30 individuals homozygous for the minor allele.

The primary analysis consisted of a case-only test for an interaction between homozygosity for the *MYH9* E1 haplotype or *APOL1* risk variants (G1/G1; G2/G2; G1/G2) and individual SNPs across the genome. Specifically, a logistic regression model was computed in cases where the binary outcome was homozygosity for *APOL1* risk SNPs or *MYH9* E1 haplotypes (versus not homozygous) and independent variables (covariates) were age, gender, first PC to account for admixture and SNP. The case-only analysis makes the strong assumption that the SNP being tested and homozygosity for the c22 variants are independent under the null hypothesis of no interaction. If the assumption of independence under the null hypothesis is met, this case-only analysis can have considerably more statistical power than the corresponding classic case-control interaction model [28]. To make the inference as robust to this assumption as possible, the test was restricted to those SNPs not on c22; note by Mendel's Law of Independent Assortment chromosomes are inherited independently and therefore the independence assumption is met. This assumption was further examined by testing for the interaction in the control sample.

As an aid to interpret the case-only interaction analysis, the corresponding classic two-locus logistic regression interaction model was computed. Here, the logistic regression model had T2DM-ESRD status as the outcome, and the predictor variables (covariates) of age, gender, PC, the SNP, an indicator variable for two *APOL1* risk variants or *MYH9* E1 haplotype homozygosity and the centered cross-product of the SNP and indicator for c22 risk variant homozygosity. Here we mean the standard logistic regression model for two predictor variables (say X_1_ and X_2_) with their interaction term, a centered cross-product (e.g., Z) to reduce collinearity/correlation among the variables. Specifically, we would write this model as: 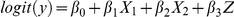; where, X_1_ is the SNP and X_2_ is the indicator variable for the APOL1/MYH9 haplotype (see below), respectively and Z is the center cross-product defined as 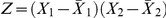. The variable Z is defined in this way to reduce the collinearity or correlation among the predictors for better estimation properties. The indicator variable is a binary variable that codes an individual as either 0 or 1, depending on the characteristic of interest. Here, the indicator variable was 1 if the person was homozygous for the *APOL1*/*MYH9* haplotype (easily determinable as it is a recessive model and phase is unambiguous) and 0 if they were not homozygous for these risk haplotypes. This binary (0, 1) variable was included in the logistic regression model. For the case-only analysis, this indicator variable was the outcome in the logistic regression analysis and for the classic two-locus interaction logistic regression models it was one of the predictor variables.

Subsequent analyses stratified by homozygosity at the *MYH9* E1 haplotype and *APOL1* risk variants. A logistic regression model was computed in individuals homozygous for c22 variants, where T2DM-ESRD status was the outcome and the independent variables (covariates) in the model included age, gender, the first PC and the SNP of interest. The analysis was repeated for individuals not homozygous for c22 variants and the test for homogeneity of the odds ratio was computed. Analyses in the replication cohorts paralleled those in the discovery cohort.

To determine whether associated SNPs from analyses contrasting individuals with T2DM-ESRD to those without diabetes were DN-associated or T2DM-associated, allele frequencies were compared between AA with T2DM lacking nephropathy to those in the combined T2DM-ESRD case groups and the combined non-diabetic, non-nephropathy control groups.

Assuming a recessive model for the *MYH9* and *APOL1* risk variants with main effect OR = 1.5, haplotype frequency of 0.64, and an additive genetic model for the *FRMD3* SNPs having no main effect (OR = 1.0) with minor allele frequency of 0.32, then with a type 1 error rate of α = 1^−10^, we have 0.50 power to detect an OR = 2.05 and 0.80 power to detect an OR = 2.34.

## Supporting Information

Figure S1 P-P Plots for unconditional diabetic nephropathy GWAS, Case-Only Analysis, and the-Two-Locus Interaction Logistic Regression Model. The three P-P Plots (left to right) for the original GWAS (McDonough et al., 2011), the Case-Only Analysis and the Two-Locus Interaction Logistic Regression Model.
(DOCX)

Table S1 Inflation factors (λ) for the three genome-wide scans of these data.
(DOCX)

Table S2 Top 100 interactive SNPs, sorted by additive model P- values.
(DOCX)

Table S3 FRMD3 SNP allele frequencies by *MYH9* risk haplotype status (letter in brackets reflects allele).
(DOCX)

Table S4 FRMD3 SNP allele frequencies by *APOL1* risk allele status (letter in brackets reflects allele).
(DOCX)

## References

[1] Kopp JB, Smith MW, Nelson GW, Johnson RC, Freedman BI (2008). MYH9 is a major-effect risk gene for focal segmental glomerulosclerosis.. Nat Genet.

[2] Kao WH, Klag MJ, Meoni LA, Reich D, Berthier-Schaad Y (2008). MYH9 is associated with nondiabetic end-stage renal disease in African Americans.. Nat Genet.

[3] Freedman BI, Hicks PJ, Bostrom MA, Cunningham ME, Liu Y (2009). Polymorphisms in the non-muscle myosin heavy chain 9 gene (MYH9) are strongly associated with end-stage renal disease historically attributed to hypertension in African Americans.. Kidney Int.

[4] Freedman BI, Hicks PJ, Bostrom MA, Comeau ME, Divers J (2009). Non-muscle myosin heavy chain 9 gene MYH9 associations in African Americans with clinically diagnosed type 2 diabetes mellitus-associated ESRD.. Nephrol Dial Transplant.

[5] Freedman BI, Kopp JB, Winkler CA, Nelson GW, Rao DC (2009). Polymorphisms in the nonmuscle myosin heavy chain 9 gene (MYH9) are associated with albuminuria in hypertensive African Americans: the HyperGEN study.. Am J Nephrol.

[6] Reeves-Daniel AM, Iskandar SS, Bowden DW, Bostrom MA, Hicks PJ (2010). Is Collapsing C1q Nephropathy Another MYH9-Associated Kidney Disease? A Case Report.. American Journal of Kidney Diseases.

[7] Pattaro C, Aulchenko YS, Isaacs A, Vitart V, Hayward C (2009). Genome-wide linkage analysis of serum creatinine in three isolated European populations.. Kidney Int.

[8] Genovese G, Friedman DJ, Ross MD, Lecordier L, Uzureau P (2010). Association of trypanolytic ApoL1 variants with kidney disease in African Americans.. Science.

[9] Tzur S, Rosset S, Shemer R, Yudkovsky G, Selig S (2010). Missense mutations in the APOL1 gene are highly associated with end stage kidney disease risk previously attributed to the MYH9 gene.. Hum Genet.

[10] Freedman BI, Kopp JB, Langefeld CD, Genovese G, Friedman DJ (2010). The Apolipoprotein L1 (APOL1) Gene and Nondiabetic Nephropathy in African Americans.. Journal of the American Society of Nephrology.

[11] Nelson GW, Freedman BI, Bowden DW, Langefeld CD, An P (2010). Dense mapping of MYH9 localizes the strongest kidney disease associations to the region of introns 13 to 15.. Hum Mol Genet.

[12] Gopalakrishnan I, Iskandar SS, Daeihagh P, Divers J, Langefeld CD (2011). Coincident idiopathic focal segmental glomerulosclerosis collapsing variant and diabetic nephropathy in an African American homozygous for MYH9 risk variants.. Hum Pathol.

[13] Divers J, Freedman BI (2010). Susceptibility genes in common complex kidney disease.. Curr Opin Nephrol Hypertens.

[14] Nunez M, Saran AM, Freedman BI (2010). Gene-gene and gene-environment interactions in HIV-associated nephropathy: A focus on the MYH9 nephropathy susceptibility gene.. Adv Chronic Kidney Dis.

[15] McDonough CW, Palmer ND, Hicks PJ, Roh BH, An SS (2011). A genome-wide association study for diabetic nephropathy genes in African Americans.. Kidney Int.

[16] Kottgen A, Glazer NL, Dehghan A, Hwang SJ, Katz R (2009). Multiple loci associated with indices of renal function and chronic kidney disease.. Nat Genet.

[17] Pezzolesi MG, Poznik GD, Mychaleckyj JC, Paterson AD, Barati MT (2009). Genome-wide association scan for diabetic nephropathy susceptibility genes in type 1 diabetes.. Diabetes.

[18] Murea M, Freedman BI (2010). Essential hypertension and risk of nephropathy: a reappraisal.. Curr Opin Nephrol Hypertens.

[19] Kottgen A, Pattaro C, Boger CA, Fuchsberger C, Olden M (2010). New loci associated with kidney function and chronic kidney disease.. Nat Genet.

[20] Maeda S, Araki SI, Babazono T, Toyoda M, Umezono T (2010). Replication study for the association between 4 loci identified by a genome-wide association study on European American subjects with type 1 diabetes and susceptibility to diabetic nephropathy in Japanese subjects with type 2 diabetes.. Diabetes.

[21] Ramez M, Blot-Chabaud M, Ciuzeaud F, Chanan S, Patterson M (2003). Distinct distribution of specific members of protein 4.1 gene family in the mouse nephron.. Kidney Int.

[22] Frame MC, Patel H, Serrels B, Lietha D, Eck MJ (2010). The FERM domain: organizing the structure and function of FAK.. Nat Rev Mol Cell Biol.

[23] Ni X, Ji C, Cao G, Cheng H, Guo L (2003). Molecular cloning and characterization of the protein 4.10 gene, a novel member of the protein 4.1 family with focal expression in ovary.. J Hum Genet.

[24] Hoover KB, Bryant PJ (2000). The genetics of the protein 4.1 family: organizers of the membrane and cytoskeleton.. Curr Opin Cell Biol.

[25] Chishti AH, Kim AC, Marfatia SM, Lutchman M, Hanspai M (1998). The FERM domain: a unique module involved in the linkage of cytoplasmic proteins to the membrane.. Trends Biochem Sci.

[26] Baines AJ (2006). A FERM-adjacent (FA) region defines a subset of the 4.1 superfamily and is a potential regulator of FERM domain function.. BMC Genomics.

[27] Tang H, Peng J, Wang P, Risch NJ (2005). Estimation of individual admixture: analytical and study design considerations.. Genet Epidemiol.

[28] Piegorsch WW, Weinberg CR, Taylor JA (1994). Non-hierarchical logistic models and case-only designs for assessing susceptibility in population-based case-control studies.. Stat Med.

