# Supplementary material for: Differential Effects of MYH9 and APOL1 Risk Variants on FRMD3 Association with Diabetic ESRD in African Americans
Source: PLoS Genet. 2011 Jun 16;7(6):e1002150. doi: 10.1371/journal.pgen.1002150 (PMC3116917; doi:10.1371/journal.pgen.1002150)
Supplement: Figure S1 — P-P Plots for unconditional diabetic nephropathy GWAS, Case-Only Analysis, and the-Two-Locus Interaction Logistic Regression Model. The three P-P Plots (left to right) for the original GWAS (McDonough et al., 2011), the Case-Only Analysis and the Two-Locus Interaction Logistic Regression Model. (DOCX) [file pgen.1002150.s001.docx]

Supplementary Figure 1. P-P Plots for unconditional diabetic nephropathy GWAS, Case-Only Analysis and the-Two-Locus Interaction Logistic Regression Model.


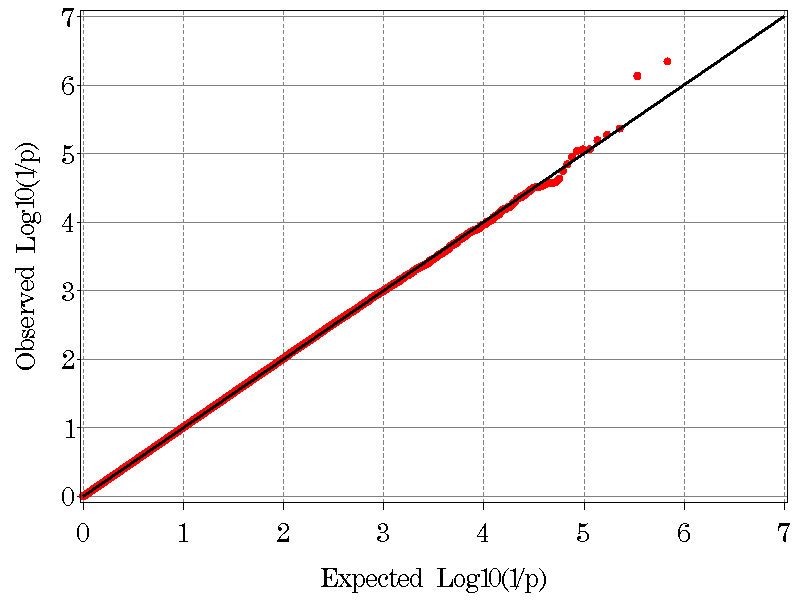


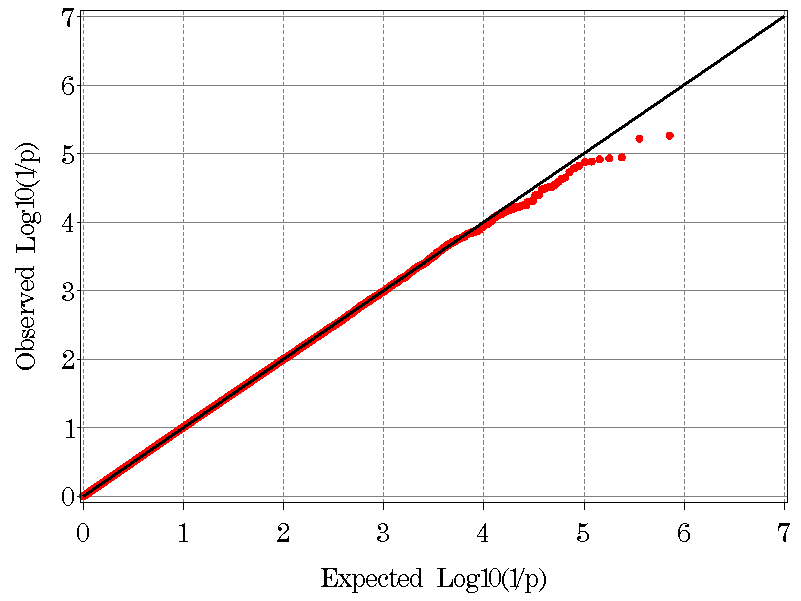


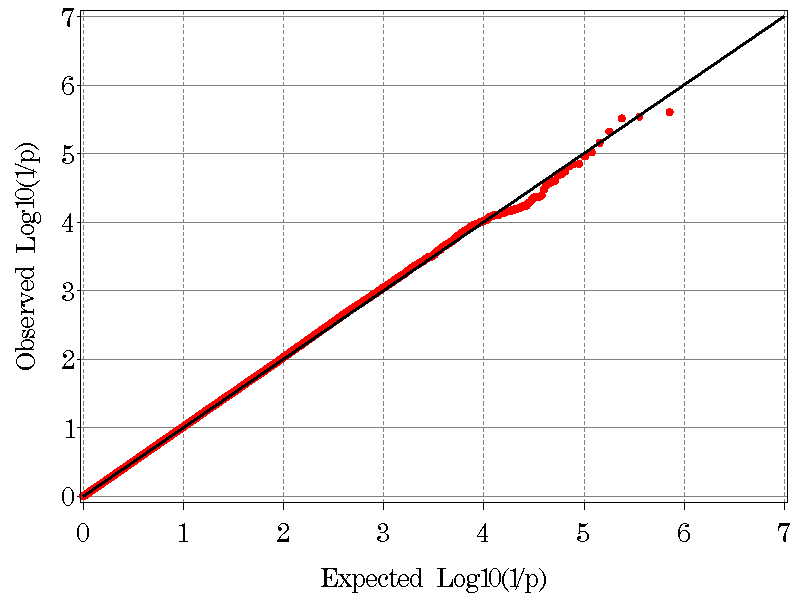


The three P-P Plots (left to right) for the original GWAS (McDonough CW et al., Kidney Int 2011), the Case-Only Analysis and the Two-Locus Interaction Logistic Regression Model
