# Supplementary material for: Differential Effects of MYH9 and APOL1 Risk Variants on FRMD3 Association with Diabetic ESRD in African Americans
Source: PLoS Genet. 2011 Jun 16;7(6):e1002150. doi: 10.1371/journal.pgen.1002150 (PMC3116917; doi:10.1371/journal.pgen.1002150)
Supplement: Table S1 — Inflation factors (λ) for the three genome-wide scans of these data. (DOCX) [file pgen.1002150.s002.docx]

Supplementary Table 1. Inflation factors (λ) for the three genome-wide scans of these data

| **Adjustment** | **GWAS*** | **Case-Only Analysis** | **Two-Locus Interaction Logistic Regression Model** |
| --- | --- | --- | --- |
| **No adjustment** | 1.13 | 1.09 | 1.01 |
| **PC1 only** | 1.04 | 1.01 | 1.01 |
| **Age, gender, PC1** | 1.02 | 1.01 | 1.01 |

* Previously published unconditional diabetic nephropathy GWAS (McDonough CW et al., Kidney Int 2011). PC – principal component.
