# Supplementary material for: Differential Effects of MYH9 and APOL1 Risk Variants on FRMD3 Association with Diabetic ESRD in African Americans
Source: PLoS Genet. 2011 Jun 16;7(6):e1002150. doi: 10.1371/journal.pgen.1002150 (PMC3116917; doi:10.1371/journal.pgen.1002150)
Supplement: Table S2 — Top 100 interactive SNPs, sorted by additive model P- values. (DOCX) [file pgen.1002150.s003.docx]

| **Supplementary Table 2: Top 100 interactive SNPs, sorted by additive model P- values** | | | | | | | | |
| --- | --- | --- | --- | --- | --- | --- | --- | --- |
| **RANK** | **SNP** | **CHR** | **POSITION** | **GENE** | **REGION** | **P-VALUE** | **Odds**  **Ratio** | **OR (95% Confidence**  **Interval)** |
| 1 | rs11196449 | [10](http://www.ncbi.nlm.nih.gov/mapview/map_search.cgi?taxid=9606&query=rs11196449%20) | 115470573 | [CASP7](http://www.ncbi.nlm.nih.gov/entrez/query.fcgi?db=gene&cmd=Retrieve&dopt=Graphics&list_uids=840) | chr10q25.3 | 5.41E-06 | 1.92 | (1.45,2.54) |
| 2 | rs7334855 | [13](http://www.ncbi.nlm.nih.gov/mapview/map_search.cgi?taxid=9606&query=rs7334855%20) | 83180656 |  | chr13q31.1 | 5.99E-06 | 0.57 | (0.45,0.73) |
| 3 | rs4709298 | [6](http://www.ncbi.nlm.nih.gov/mapview/map_search.cgi?taxid=9606&query=rs4709298%20) | 157834995 | [LOC730077,ZDHHC14](http://www.ncbi.nlm.nih.gov/entrez/query.fcgi?db=gene&cmd=Retrieve&dopt=Graphics&list_uids=730077) | chr6q25.3 | 1.17E-05 | 0.59 | (0.47,0.75) |
| 4 | rs7327372 | [13](http://www.ncbi.nlm.nih.gov/mapview/map_search.cgi?taxid=9606&query=rs7327372%20) | 91377251 | [GPC5](http://www.ncbi.nlm.nih.gov/entrez/query.fcgi?db=gene&cmd=Retrieve&dopt=Graphics&list_uids=2262) | chr13q31.3 | 1.20E-05 | 1.97 | (1.45,2.66) |
| 5 | rs9407332 | [9](http://www.ncbi.nlm.nih.gov/mapview/map_search.cgi?taxid=9606&query=rs9407332%20) | 641334 | [ANKRD15](http://www.ncbi.nlm.nih.gov/entrez/query.fcgi?db=gene&cmd=Retrieve&dopt=Graphics&list_uids=23189) | chr9p24.3 | 1.30E-05 | 2.15 | (1.52,3.03) |
| 6 | rs898040 | [6](http://www.ncbi.nlm.nih.gov/mapview/map_search.cgi?taxid=9606&query=rs898040%20) | 78408636 |  | chr6q14.1 | 1.32E-05 | 1.69 | (1.34,2.15) |
| 7 | rs10189339 | [2](http://www.ncbi.nlm.nih.gov/mapview/map_search.cgi?taxid=9606&query=rs10189339%20) | 45947265 | [PRKCE](http://www.ncbi.nlm.nih.gov/entrez/query.fcgi?db=gene&cmd=Retrieve&dopt=Graphics&list_uids=5581) | chr2p21 | 1.49E-05 | 0.64 | (0.52,0.78) |
| 8 | rs17127259 | [12](http://www.ncbi.nlm.nih.gov/mapview/map_search.cgi?taxid=9606&query=rs17127259%20) | 38589899 | [SLC2A13](http://www.ncbi.nlm.nih.gov/entrez/query.fcgi?db=gene&cmd=Retrieve&dopt=Graphics&list_uids=114134) | chr12q12 | 1.63E-05 | 1.84 | (1.39,2.43) |
| 9 | rs6046263 | [20](http://www.ncbi.nlm.nih.gov/mapview/map_search.cgi?taxid=9606&query=rs6046263%20) | 19633245 | [SLC24A3](http://www.ncbi.nlm.nih.gov/entrez/query.fcgi?db=gene&cmd=Retrieve&dopt=Graphics&list_uids=57419) | chr20p11.23 | 1.85E-05 | 1.67 | (1.32,2.11) |
| 10 | rs17002201 | [4](http://www.ncbi.nlm.nih.gov/mapview/map_search.cgi?taxid=9606&query=rs17002201%20) | 77900983 | [SHROOM3](http://www.ncbi.nlm.nih.gov/entrez/query.fcgi?db=gene&cmd=Retrieve&dopt=Graphics&list_uids=57619) | chr4q21.1 | 2.21E-05 | 0.55 | (0.42,0.72) |
| 11 | rs1446542 | [2](http://www.ncbi.nlm.nih.gov/mapview/map_search.cgi?taxid=9606&query=rs1446542%20) | 13764186 |  | chr2p24.3 | 2.33E-05 | 0.65 | (0.53,0.79) |
| 12 | rs2911613 | [1](http://www.ncbi.nlm.nih.gov/mapview/map_search.cgi?taxid=9606&query=rs2911613%20) | 84883713 | [SSX2IP](http://www.ncbi.nlm.nih.gov/entrez/query.fcgi?db=gene&cmd=Retrieve&dopt=Graphics&list_uids=117178) | chr1p22.3 | 2.59E-05 | 1.57 | (1.27,1.94) |
| 13 | rs11172591 | [12](http://www.ncbi.nlm.nih.gov/mapview/map_search.cgi?taxid=9606&query=rs11172591%20) | 57088509 |  | chr12q14.1 | 2.83E-05 | 1.59 | (1.28,1.97) |
| 14 | rs11071655 | [15](http://www.ncbi.nlm.nih.gov/mapview/map_search.cgi?taxid=9606&query=rs11071655%20) | 60215266 |  | chr15q22.2 | 3.04E-05 | 1.76 | (1.35,2.29) |
| 15 | rs17049174 | [3](http://www.ncbi.nlm.nih.gov/mapview/map_search.cgi?taxid=9606&query=rs17049174%20) | 8484498 |  | chr3p26.1 | 3.04E-05 | 1.79 | (1.36,2.35) |
| 16 | rs16884396 | [6](http://www.ncbi.nlm.nih.gov/mapview/map_search.cgi?taxid=9606&query=rs16884396%20) | 21131728 | [CDKAL1](http://www.ncbi.nlm.nih.gov/entrez/query.fcgi?db=gene&cmd=Retrieve&dopt=Graphics&list_uids=54901) | chr6p22.3 | 3.12E-05 | 0.61 | (0.48,0.77) |
| 17 | rs2466130 | [8](http://www.ncbi.nlm.nih.gov/mapview/map_search.cgi?taxid=9606&query=rs2466130%20) | 97251446 |  | chr8q22.1 | 3.23E-05 | 2.01 | (1.45,2.8) |
| 18 | rs1545606 | [4](http://www.ncbi.nlm.nih.gov/mapview/map_search.cgi?taxid=9606&query=rs1545606%20) | 25484360 |  | chr4p15.2 | 3.35E-05 | 2.04 | (1.46,2.85) |
| 19 | rs1341795 | [1](http://www.ncbi.nlm.nih.gov/mapview/map_search.cgi?taxid=9606&query=rs1341795%20) | 69732725 |  | chr1p31.1 | 3.94E-05 | 0.61 | (0.48,0.77) |
| 20 | rs2181083 | [14](http://www.ncbi.nlm.nih.gov/mapview/map_search.cgi?taxid=9606&query=rs2181083%20) | 50363457 | [NIN](http://www.ncbi.nlm.nih.gov/entrez/query.fcgi?db=gene&cmd=Retrieve&dopt=Graphics&list_uids=51199) | chr14q22.1 | 4.01E-05 | 1.65 | (1.3,2.1) |
| 21 | rs4435859 | [5](http://www.ncbi.nlm.nih.gov/mapview/map_search.cgi?taxid=9606&query=rs4435859%20) | 124155620 |  | chr5q23.2 | 4.04E-05 | 1.53 | (1.25,1.88) |
| 22 | rs9826624 | [3](http://www.ncbi.nlm.nih.gov/mapview/map_search.cgi?taxid=9606&query=rs9826624%20) | 23125313 |  | chr3p24.3 | 4.82E-05 | 1.52 | (1.24,1.85) |
| 23 | rs6748268 | [2](http://www.ncbi.nlm.nih.gov/mapview/map_search.cgi?taxid=9606&query=rs6748268%20) | 71006359 | [VAX2](http://www.ncbi.nlm.nih.gov/entrez/query.fcgi?db=gene&cmd=Retrieve&dopt=Graphics&list_uids=25806) | chr2p13.3 | 4.96E-05 | 1.64 | (1.29,2.09) |
| 24 | rs12434641 | [14](http://www.ncbi.nlm.nih.gov/mapview/map_search.cgi?taxid=9606&query=rs12434641%20) | 92427865 |  | chr14q32.12 | 4.97E-05 | 1.62 | (1.28,2.05) |
| 25 | rs4352152 | [19](http://www.ncbi.nlm.nih.gov/mapview/map_search.cgi?taxid=9606&query=rs4352152%20) | 48159696 |  | chr19q13.31 | 5.02E-05 | 1.63 | (1.29,2.06) |
| 26 | rs7107396 | [11](http://www.ncbi.nlm.nih.gov/mapview/map_search.cgi?taxid=9606&query=rs7107396%20) | 122701419 |  | chr11q24.1 | 5.65E-05 | 1.59 | (1.27,1.99) |
| 27 | rs7191955 | [16](http://www.ncbi.nlm.nih.gov/mapview/map_search.cgi?taxid=9606&query=rs7191955%20) | 20146088 |  | chr16p12.3 | 5.66E-05 | 0.67 | (0.55,0.81) |
| 28 | rs2273800 | [14](http://www.ncbi.nlm.nih.gov/mapview/map_search.cgi?taxid=9606&query=rs2273800%20) | 99917461 | [WDR25](http://www.ncbi.nlm.nih.gov/entrez/query.fcgi?db=gene&cmd=Retrieve&dopt=Graphics&list_uids=79446) | chr14q32.2 | 5.68E-05 | 0.66 | (0.53,0.81) |
| 29 | rs539674 | [8](http://www.ncbi.nlm.nih.gov/mapview/map_search.cgi?taxid=9606&query=rs539674%20) | 102553663 |  | chr8q22.3 | 5.87E-05 | 1.51 | (1.24,1.85) |
| 30 | rs3742387 | [14](http://www.ncbi.nlm.nih.gov/mapview/map_search.cgi?taxid=9606&query=rs3742387%20) | 99917684 | [WDR25](http://www.ncbi.nlm.nih.gov/entrez/query.fcgi?db=gene&cmd=Retrieve&dopt=Graphics&list_uids=79446) | chr14q32.2 | 5.91E-05 | 0.66 | (0.54,0.81) |
| 31 | rs4370836 | [10](http://www.ncbi.nlm.nih.gov/mapview/map_search.cgi?taxid=9606&query=rs4370836%20) | 2904754 |  | chr10p15.3 | 5.96E-05 | 0.59 | (0.46,0.77) |
| 32 | rs4382808 | [10](http://www.ncbi.nlm.nih.gov/mapview/map_search.cgi?taxid=9606&query=rs4382808%20) | 2906559 |  | chr10p15.3 | 5.96E-05 | 0.59 | (0.46,0.77) |
| 33 | rs4413550 | [5](http://www.ncbi.nlm.nih.gov/mapview/map_search.cgi?taxid=9606&query=rs4413550%20) | 178005242 |  | chr5q35.3 | 6.19E-05 | 0.66 | (0.54,0.81) |
| 34 | rs349549 | [3](http://www.ncbi.nlm.nih.gov/mapview/map_search.cgi?taxid=9606&query=rs349549%20) | 141741211 | [CLSTN2](http://www.ncbi.nlm.nih.gov/entrez/query.fcgi?db=gene&cmd=Retrieve&dopt=Graphics&list_uids=64084) | chr3q23 | 6.21E-05 | 0.65 | (0.52,0.8) |
| 35 | rs3763608 | [9](http://www.ncbi.nlm.nih.gov/mapview/map_search.cgi?taxid=9606&query=rs3763608%20) | 70837196 |  | chr9q21.11 | 6.47E-05 | 0.59 | (0.45,0.76) |
| 36 | rs10166439 | [2](http://www.ncbi.nlm.nih.gov/mapview/map_search.cgi?taxid=9606&query=rs10166439%20) | 169471807 | [G6PC2](http://www.ncbi.nlm.nih.gov/entrez/query.fcgi?db=gene&cmd=Retrieve&dopt=Graphics&list_uids=57818) | chr2q31.1 | 6.51E-05 | 1.58 | (1.26,1.98) |
| 37 | rs6107706 | [20](http://www.ncbi.nlm.nih.gov/mapview/map_search.cgi?taxid=9606&query=rs6107706%20) | 583055 | [SRXN1](http://www.ncbi.nlm.nih.gov/entrez/query.fcgi?db=gene&cmd=Retrieve&dopt=Graphics&list_uids=140809) | chr20p13 | 6.57E-05 | 0.51 | (0.37,0.71) |
| 38 | rs10789294 | [1](http://www.ncbi.nlm.nih.gov/mapview/map_search.cgi?taxid=9606&query=rs10789294%20) | 69712128 |  | chr1p31.1 | 6.72E-05 | 0.63 | (0.51,0.79) |
| 39 | rs13431495 | [2](http://www.ncbi.nlm.nih.gov/mapview/map_search.cgi?taxid=9606&query=rs13431495%20) | 65829259 |  | chr2p14 | 6.74E-05 | 1.62 | (1.28,2.05) |
| 40 | rs4802074 | [19](http://www.ncbi.nlm.nih.gov/mapview/map_search.cgi?taxid=9606&query=rs4802074%20) | 45539922 | [C19orf47](http://www.ncbi.nlm.nih.gov/entrez/query.fcgi?db=gene&cmd=Retrieve&dopt=Graphics&list_uids=126526) | chr19q13.2 | 6.75E-05 | 1.61 | (1.28,2.04) |
| 41 | rs2309393 | [9](http://www.ncbi.nlm.nih.gov/mapview/map_search.cgi?taxid=9606&query=rs2309393%20) | 70865909 | [FXN](http://www.ncbi.nlm.nih.gov/entrez/query.fcgi?db=gene&cmd=Retrieve&dopt=Graphics&list_uids=2395) | chr9q21.11 | 7.10E-05 | 0.6 | (0.46,0.77) |
| 42 | rs10889921 | [1](http://www.ncbi.nlm.nih.gov/mapview/map_search.cgi?taxid=9606&query=rs10889921%20) | 71818215 | [NEGR1](http://www.ncbi.nlm.nih.gov/entrez/query.fcgi?db=gene&cmd=Retrieve&dopt=Graphics&list_uids=257194) | chr1p31.1 | 7.25E-05 | 0.56 | (0.42,0.74) |
| 43 | rs11608184 | [11](http://www.ncbi.nlm.nih.gov/mapview/map_search.cgi?taxid=9606&query=rs11608184%20) | 106125221 | [GUCY1A2](http://www.ncbi.nlm.nih.gov/entrez/query.fcgi?db=gene&cmd=Retrieve&dopt=Graphics&list_uids=2977) | chr11q22.3 | 7.48E-05 | 1.97 | (1.41,2.75) |
| 44 | rs2436893 | [8](http://www.ncbi.nlm.nih.gov/mapview/map_search.cgi?taxid=9606&query=rs2436893%20) | 103643840 |  | chr8q22.3 | 7.49E-05 | 1.5 | (1.23,1.84) |
| 45 | rs10921075 | [1](http://www.ncbi.nlm.nih.gov/mapview/map_search.cgi?taxid=9606&query=rs10921075%20) | 186945541 |  | chr1q31.1 | 7.50E-05 | 0.65 | (0.52,0.8) |
| 46 | rs1927369 | [13](http://www.ncbi.nlm.nih.gov/mapview/map_search.cgi?taxid=9606&query=rs1927369%20) | 101933445 |  | chr13q33.1 | 7.65E-05 | 0.66 | (0.54,0.81) |
| 47 | rs7164376 | [15](http://www.ncbi.nlm.nih.gov/mapview/map_search.cgi?taxid=9606&query=rs7164376%20) | 86197104 |  | chr15q25.3 | 7.67E-05 | 0.66 | (0.53,0.81) |
| 48 | rs1952142 | [1](http://www.ncbi.nlm.nih.gov/mapview/map_search.cgi?taxid=9606&query=rs1952142%20) | 69721443 |  | chr1p31.1 | 7.92E-05 | 0.62 | (0.49,0.79) |
| 49 | rs9827163 | [3](http://www.ncbi.nlm.nih.gov/mapview/map_search.cgi?taxid=9606&query=rs9827163%20) | 23125478 |  | chr3p24.3 | 8.01E-05 | 1.48 | (1.22,1.79) |
| 50 | rs9407334 | [9](http://www.ncbi.nlm.nih.gov/mapview/map_search.cgi?taxid=9606&query=rs9407334%20) | 641462 | [ANKRD15](http://www.ncbi.nlm.nih.gov/entrez/query.fcgi?db=gene&cmd=Retrieve&dopt=Graphics&list_uids=23189) | chr9p24.3 | 8.26E-05 | 2.03 | (1.43,2.9) |
| 51 | rs4814885 | [20](http://www.ncbi.nlm.nih.gov/mapview/map_search.cgi?taxid=9606&query=rs4814885%20) | 19639447 | [SLC24A3](http://www.ncbi.nlm.nih.gov/entrez/query.fcgi?db=gene&cmd=Retrieve&dopt=Graphics&list_uids=57419) | chr20p11.23 | 8.29E-05 | 1.55 | (1.25,1.92) |
| 52 | rs9328127 | [6](http://www.ncbi.nlm.nih.gov/mapview/map_search.cgi?taxid=9606&query=rs9328127%20) | 274870 | [DUSP22](http://www.ncbi.nlm.nih.gov/entrez/query.fcgi?db=gene&cmd=Retrieve&dopt=Graphics&list_uids=56940) | chr6p25.3 | 8.48E-05 | 0.6 | (0.47,0.78) |
| 53 | rs12827379 | [12](http://www.ncbi.nlm.nih.gov/mapview/map_search.cgi?taxid=9606&query=rs12827379%20) | 11862891 | [ETV6](http://www.ncbi.nlm.nih.gov/entrez/query.fcgi?db=gene&cmd=Retrieve&dopt=Graphics&list_uids=2120) | chr12p13.2 | 8.54E-05 | 1.55 | (1.25,1.94) |
| 54 | rs252170 | [12](http://www.ncbi.nlm.nih.gov/mapview/map_search.cgi?taxid=9606&query=rs252170%20) | 95815980 |  | chr12q23.1 | 8.95E-05 | 1.6 | (1.27,2.03) |
| 55 | rs1892302 | [10](http://www.ncbi.nlm.nih.gov/mapview/map_search.cgi?taxid=9606&query=rs1892302%20) | 12486579 | [CAMK1D](http://www.ncbi.nlm.nih.gov/entrez/query.fcgi?db=gene&cmd=Retrieve&dopt=Graphics&list_uids=57118) | chr10p13 | 9.38E-05 | 1.51 | (1.23,1.85) |
| 56 | rs12184638 | [13](http://www.ncbi.nlm.nih.gov/mapview/map_search.cgi?taxid=9606&query=rs12184638%20) | 91427859 | [GPC5](http://www.ncbi.nlm.nih.gov/entrez/query.fcgi?db=gene&cmd=Retrieve&dopt=Graphics&list_uids=2262) | chr13q31.3 | 9.41E-05 | 2.11 | (1.45,3.06) |
| 57 | rs7812995 | [8](http://www.ncbi.nlm.nih.gov/mapview/map_search.cgi?taxid=9606&query=rs7812995%20) | 74616155 |  | chr8q21.11 | 9.65E-05 | 1.78 | (1.33,2.38) |
| 58 | rs1992932 | [2](http://www.ncbi.nlm.nih.gov/mapview/map_search.cgi?taxid=9606&query=rs1992932%20) | 45941378 | [PRKCE](http://www.ncbi.nlm.nih.gov/entrez/query.fcgi?db=gene&cmd=Retrieve&dopt=Graphics&list_uids=5581) | chr2p21 | 9.73E-05 | 0.67 | (0.54,0.82) |
| 59 | rs7017095 | [8](http://www.ncbi.nlm.nih.gov/mapview/map_search.cgi?taxid=9606&query=rs7017095%20) | 108296238 |  | chr8q23.1 | 9.97E-05 | 1.6 | (1.26,2.02) |
| 60 | rs11988877 | [8](http://www.ncbi.nlm.nih.gov/mapview/map_search.cgi?taxid=9606&query=rs11988877%20) | 107027362 |  | chr8q23.1 | 1.01E-04 | 1.55 | (1.24,1.93) |
| 61 | rs16823704 | [2](http://www.ncbi.nlm.nih.gov/mapview/map_search.cgi?taxid=9606&query=rs16823704%20) | 183375479 |  | chr2q32.1 | 1.03E-04 | 1.89 | (1.37,2.62) |
| 62 | rs1353449 | [8](http://www.ncbi.nlm.nih.gov/mapview/map_search.cgi?taxid=9606&query=rs1353449%20) | 107012422 |  | chr8q23.1 | 1.04E-04 | 1.59 | (1.26,2.01) |
| 63 | rs925197 | [5](http://www.ncbi.nlm.nih.gov/mapview/map_search.cgi?taxid=9606&query=rs925197%20) | 174871821 | [SFXN1](http://www.ncbi.nlm.nih.gov/entrez/query.fcgi?db=gene&cmd=Retrieve&dopt=Graphics&list_uids=94081) | chr5q35.2 | 1.07E-04 | 0.67 | (0.55,0.82) |
| 64 | rs12798695 | [11](http://www.ncbi.nlm.nih.gov/mapview/map_search.cgi?taxid=9606&query=rs12798695%20) | 106135967 | [GUCY1A2](http://www.ncbi.nlm.nih.gov/entrez/query.fcgi?db=gene&cmd=Retrieve&dopt=Graphics&list_uids=2977) | chr11q22.3 | 1.07E-04 | 1.83 | (1.35,2.49) |
| 65 | rs1123753 | [6](http://www.ncbi.nlm.nih.gov/mapview/map_search.cgi?taxid=9606&query=rs1123753%20) | 169302765 |  | chr6q27 | 1.09E-04 | 0.67 | (0.55,0.82) |
| 66 | rs969531 | [19](http://www.ncbi.nlm.nih.gov/mapview/map_search.cgi?taxid=9606&query=rs969531%20) | 45470707 | [AKT2](http://www.ncbi.nlm.nih.gov/entrez/query.fcgi?db=gene&cmd=Retrieve&dopt=Graphics&list_uids=208) | chr19q13.2 | 1.09E-04 | 1.59 | (1.26,2.02) |
| 67 | rs1254152 | [10](http://www.ncbi.nlm.nih.gov/mapview/map_search.cgi?taxid=9606&query=rs1254152%20) | 122572604 |  | chr10q26.12 | 1.14E-04 | 0.68 | (0.56,0.83) |
| 68 | rs10910960 | [1](http://www.ncbi.nlm.nih.gov/mapview/map_search.cgi?taxid=9606&query=rs10910960%20) | 179798956 | [CACNA1E](http://www.ncbi.nlm.nih.gov/entrez/query.fcgi?db=gene&cmd=Retrieve&dopt=Graphics&list_uids=777) | chr1q25.3 | 1.15E-04 | 0.5 | (0.36,0.71) |
| 69 | rs17041111 | [4](http://www.ncbi.nlm.nih.gov/mapview/map_search.cgi?taxid=9606&query=rs17041111%20) | 111113442 | [EGF](http://www.ncbi.nlm.nih.gov/entrez/query.fcgi?db=gene&cmd=Retrieve&dopt=Graphics&list_uids=1950) | chr4q25 | 1.18E-04 | 2.07 | (1.43,3) |
| 70 | rs10280425 | [7](http://www.ncbi.nlm.nih.gov/mapview/map_search.cgi?taxid=9606&query=rs10280425%20) | 147876736 |  | chr7q36.1 | 1.18E-04 | 1.78 | (1.33,2.39) |
| 71 | rs429217 | [15](http://www.ncbi.nlm.nih.gov/mapview/map_search.cgi?taxid=9606&query=rs429217%20) | 77820682 |  | chr15q25.1 | 1.18E-04 | 1.58 | (1.25,1.99) |
| 72 | rs7655852 | [4](http://www.ncbi.nlm.nih.gov/mapview/map_search.cgi?taxid=9606&query=rs7655852%20) | 5109881 | [STK32B](http://www.ncbi.nlm.nih.gov/entrez/query.fcgi?db=gene&cmd=Retrieve&dopt=Graphics&list_uids=55351) | chr4p16.2 | 1.19E-04 | 0.68 | (0.56,0.83) |
| 73 | rs17249444 | [14](http://www.ncbi.nlm.nih.gov/mapview/map_search.cgi?taxid=9606&query=rs17249444%20) | 67171208 | [ARG2](http://www.ncbi.nlm.nih.gov/entrez/query.fcgi?db=gene&cmd=Retrieve&dopt=Graphics&list_uids=384) | chr14q24.1 | 1.25E-04 | 0.66 | (0.53,0.82) |
| 74 | rs7313032 | [12](http://www.ncbi.nlm.nih.gov/mapview/map_search.cgi?taxid=9606&query=rs7313032%20) | 122738685 | [TCTN2](http://www.ncbi.nlm.nih.gov/entrez/query.fcgi?db=gene&cmd=Retrieve&dopt=Graphics&list_uids=79867) | chr12q24.31 | 1.26E-04 | 0.68 | (0.55,0.83) |
| 75 | rs1342553 | [1](http://www.ncbi.nlm.nih.gov/mapview/map_search.cgi?taxid=9606&query=rs1342553%20) | 187447617 |  | chr1q31.1 | 1.27E-04 | 0.64 | (0.51,0.8) |
| 76 | rs7225158 | [17](http://www.ncbi.nlm.nih.gov/mapview/map_search.cgi?taxid=9606&query=rs7225158%20) | 10036179 | [GAS7](http://www.ncbi.nlm.nih.gov/entrez/query.fcgi?db=gene&cmd=Retrieve&dopt=Graphics&list_uids=8522) | chr17p13.1 | 1.31E-04 | 1.45 | (1.2,1.76) |
| 77 | rs2952017 | [8](http://www.ncbi.nlm.nih.gov/mapview/map_search.cgi?taxid=9606&query=rs2952017%20) | 26079409 |  | chr8p21.2 | 1.34E-04 | 1.46 | (1.2,1.77) |
| 78 | rs16860688 | [1](http://www.ncbi.nlm.nih.gov/mapview/map_search.cgi?taxid=9606&query=rs16860688%20) | 166730493 |  | chr1q24.2 | 1.34E-04 | 1.58 | (1.25,1.99) |
| 79 | rs2449066 | [3](http://www.ncbi.nlm.nih.gov/mapview/map_search.cgi?taxid=9606&query=rs2449066%20) | 100219961 |  | chr3q12.1 | 1.36E-04 | 0.65 | (0.53,0.81) |
| 80 | rs721709 | [12](http://www.ncbi.nlm.nih.gov/mapview/map_search.cgi?taxid=9606&query=rs721709%20) | 38995681 | [LRRK2](http://www.ncbi.nlm.nih.gov/entrez/query.fcgi?db=gene&cmd=Retrieve&dopt=Graphics&list_uids=120892) | chr12q12 | 1.36E-04 | 1.57 | (1.25,1.99) |
| 81 | rs4245120 | [11](http://www.ncbi.nlm.nih.gov/mapview/map_search.cgi?taxid=9606&query=rs4245120%20) | 132995127 |  | chr11q25 | 1.37E-04 | 0.61 | (0.48,0.79) |
| 82 | rs323598 | [18](http://www.ncbi.nlm.nih.gov/mapview/map_search.cgi?taxid=9606&query=rs323598%20) | 36732812 |  | chr18q12.3 | 1.37E-04 | 0.6 | (0.46,0.78) |
| 83 | rs11099590 | [4](http://www.ncbi.nlm.nih.gov/mapview/map_search.cgi?taxid=9606&query=rs11099590%20) | 84443523 | [HPSE](http://www.ncbi.nlm.nih.gov/entrez/query.fcgi?db=gene&cmd=Retrieve&dopt=Graphics&list_uids=10855) | chr4q21.23 | 1.37E-04 | 0.62 | (0.49,0.79) |
| 84 | rs11199581 | [10](http://www.ncbi.nlm.nih.gov/mapview/map_search.cgi?taxid=9606&query=rs11199581%20) | 122571387 |  | chr10q26.12 | 1.37E-04 | 1.52 | (1.22,1.88) |
| 85 | rs4900409 | [14](http://www.ncbi.nlm.nih.gov/mapview/map_search.cgi?taxid=9606&query=rs4900409%20) | 98126365 |  | chr14q32.2 | 1.38E-04 | 0.68 | (0.55,0.83) |
| 86 | rs4881388 | [10](http://www.ncbi.nlm.nih.gov/mapview/map_search.cgi?taxid=9606&query=rs4881388%20) | 5091105 |  | chr10p15.1 | 1.39E-04 | 0.67 | (0.55,0.82) |
| 87 | rs1976011 | [1](http://www.ncbi.nlm.nih.gov/mapview/map_search.cgi?taxid=9606&query=rs1976011%20) | 236435630 |  | chr1q43 | 1.39E-04 | 1.48 | (1.21,1.82) |
| 88 | rs10867977 | [9](http://www.ncbi.nlm.nih.gov/mapview/map_search.cgi?taxid=9606&query=rs10867977%20) | 85097337 | [FRMD3](http://www.ncbi.nlm.nih.gov/entrez/query.fcgi?db=gene&cmd=Retrieve&dopt=Graphics&list_uids=257019) | chr9q21.32 | 1.40E-04 | 0.67 | (0.54,0.82) |
| 89 | rs7013082 | [8](http://www.ncbi.nlm.nih.gov/mapview/map_search.cgi?taxid=9606&query=rs7013082%20) | 107012964 |  | chr8q23.1 | 1.41E-04 | 1.58 | (1.25,1.99) |
| 90 | rs12155302 | [7](http://www.ncbi.nlm.nih.gov/mapview/map_search.cgi?taxid=9606&query=rs12155302%20) | 47567608 |  | chr7p12.3 | 1.43E-04 | 1.64 | (1.27,2.12) |
| 91 | rs2191586 | [2](http://www.ncbi.nlm.nih.gov/mapview/map_search.cgi?taxid=9606&query=rs2191586%20) | 107172083 |  | chr2q12.3 | 1.44E-04 | 0.67 | (0.55,0.83) |
| 92 | rs11840214 | [13](http://www.ncbi.nlm.nih.gov/mapview/map_search.cgi?taxid=9606&query=rs11840214%20) | 105960945 | [EFNB2](http://www.ncbi.nlm.nih.gov/entrez/query.fcgi?db=gene&cmd=Retrieve&dopt=Graphics&list_uids=1948) | chr13q33.3 | 1.44E-04 | 1.44 | (1.19,1.73) |
| 93 | rs10152907 | [15](http://www.ncbi.nlm.nih.gov/mapview/map_search.cgi?taxid=9606&query=rs10152907%20) | 52679009 | [UNC13C](http://www.ncbi.nlm.nih.gov/entrez/query.fcgi?db=gene&cmd=Retrieve&dopt=Graphics&list_uids=440279) | chr15q21.3 | 1.44E-04 | 1.48 | (1.21,1.81) |
| 94 | rs2160512 | [12](http://www.ncbi.nlm.nih.gov/mapview/map_search.cgi?taxid=9606&query=rs2160512%20) | 16410288 |  | chr12p12.3 | 1.45E-04 | 0.65 | (0.52,0.81) |
| 95 | rs7958693 | [12](http://www.ncbi.nlm.nih.gov/mapview/map_search.cgi?taxid=9606&query=rs7958693%20) | 122728704 | [TCTN2](http://www.ncbi.nlm.nih.gov/entrez/query.fcgi?db=gene&cmd=Retrieve&dopt=Graphics&list_uids=79867) | chr12q24.31 | 1.46E-04 | 0.68 | (0.56,0.83) |
| 96 | rs7822763 | [8](http://www.ncbi.nlm.nih.gov/mapview/map_search.cgi?taxid=9606&query=rs7822763%20) | 26080185 |  | chr8p21.2 | 1.47E-04 | 0.68 | (0.56,0.83) |
| 97 | rs11705857 | [3](http://www.ncbi.nlm.nih.gov/mapview/map_search.cgi?taxid=9606&query=rs11705857%20) | 193734392 | [FGF12](http://www.ncbi.nlm.nih.gov/entrez/query.fcgi?db=gene&cmd=Retrieve&dopt=Graphics&list_uids=2257) | chr3q28 | 1.48E-04 | 0.62 | (0.48,0.79) |
| 98 | rs10791298 | [11](http://www.ncbi.nlm.nih.gov/mapview/map_search.cgi?taxid=9606&query=rs10791298%20) | 132996155 |  | chr11q25 | 1.48E-04 | 0.62 | (0.48,0.79) |
| 99 | rs942280 | [9](http://www.ncbi.nlm.nih.gov/mapview/map_search.cgi?taxid=9606&query=rs942280%20) | 85095682 | [FRMD3](http://www.ncbi.nlm.nih.gov/entrez/query.fcgi?db=gene&cmd=Retrieve&dopt=Graphics&list_uids=257019) | chr9q21.32 | 1.48E-04 | 0.67 | (0.54,0.82) |
| 100 | rs6856498 | [4](http://www.ncbi.nlm.nih.gov/mapview/map_search.cgi?taxid=9606&query=rs6856498%20) | 178436481 |  | chr4q34.3 | 1.49E-04 | 0.69 | (0.57,0.84) |
