# Supplementary material for: Differential Effects of MYH9 and APOL1 Risk Variants on FRMD3 Association with Diabetic ESRD in African Americans
Source: PLoS Genet. 2011 Jun 16;7(6):e1002150. doi: 10.1371/journal.pgen.1002150 (PMC3116917; doi:10.1371/journal.pgen.1002150)
Supplement: Table S3 — FRMD3 SNP allele frequencies by MYH9 risk haplotype status (letter in brackets reflects allele). (DOCX) [file pgen.1002150.s004.docx]

| Supplementary Table 3: *FRMD3* SNP allele frequencies by *MYH9* risk haplotype status (letter in brackets reflects allele) | | | | |  |
| --- | --- | --- | --- | --- | --- |
| ***FRMD3* SNP** | ***MYH9* E1 risk**  **haplotype count** | **Allele Frequency (Count)** | | | |
|  |  | **Non-diabetic**  **controls** | **T2DM non-**  **nephropathy controls** | **T2DM-ESRD cases** | |
| **rs2378658 (C)** | 0 | 0.33 (N=95) | 0.50 (N=20) | 0.28 (N=60) | |
|  | 1 | 0.32 (N=948) | 0.33 (N=305) | 0.36 (N=830) | |
|  | 2 | 0.31 (N=551) | 0.31 (N=169) | 0.27 (N=622) | |
| **rs1535753 (T)** | 0 | 0.33 (N=95) | 0.50 (N=20) | 0.28 (N=60) | |
|  | 1 | 0.32 (N=946) | 0.32 (N=304) | 0.36 (N=827) | |
|  | 2 | 0.31 (N=547) | 0.31 (N=170) | 0.27 (N=623) | |
| **rs942283 (C)** | 0 | 0.33 (N=95) | 0.50 (N=20) | 0.28 (N=60) | |
|  | 1 | 0.32 (N=947) | 0.33 (N=307) | 0.36 (N=830) | |
|  | 2 | 0.31 (N=550) | 0.31 (N=169) | 0.27 (N=626) | |
| **rs942280 (G)** | 0 | 0.33 (N=95) | 0.50 (N=20) | 0.29 (N=60) | |
|  | 1 | 0.32 (N=946) | 0.33 (N=301) | 0.37 (N=830) | |
|  | 2 | 0.32 (N=548) | 0.30 (N=169) | 0.27 (N=624) | |
| **rs942278 (T)** | 0 | 0.33 (N=95) | 0.45 (N=20) | 0.27 (N=59) | |
|  | 1 | 0.31 (N=946) | 0.32 (N=303) | 0.37 (N=828) | |
|  | 2 | 0.32 (N=547) | 0.32 (N=170) | 0.28 (N=622) | |
