# Supplementary material for: Differential Effects of MYH9 and APOL1 Risk Variants on FRMD3 Association with Diabetic ESRD in African Americans
Source: PLoS Genet. 2011 Jun 16;7(6):e1002150. doi: 10.1371/journal.pgen.1002150 (PMC3116917; doi:10.1371/journal.pgen.1002150)
Supplement: Table S4 — FRMD3 SNP allele frequencies by APOL1 risk allele status (letter in brackets reflects allele). (DOCX) [file pgen.1002150.s005.docx]

| Supplementary Table 4: *FRMD3* SNP allele frequencies by *APOL1* risk allele status (letter in brackets reflects allele) | | | | |
| --- | --- | --- | --- | --- |
| **SNP** | ***APOL1* G1/G2**  **risk allele count** | **Allele Frequency (Count)** | | |
|  |  | **Non-diabetic**  **controls** | **T2DM non-**  **nephropathy controls** | **T2DM-ESRD cases** |
| **rs2378658 (C)** | 0 | 0.31 (N=631) | 0.31 (N=218) | 0.34 (N=513) |
|  | 1 | 0.32 (N=653) | 0.34 (N=228) | 0.33 (N=692) |
|  | 2 | 0.33 (N=215) | 0.37 (N=55) | 0.27 (N=325) |
| **rs1535753 (T)** | 0 | 0.31 (N=632) | 0.31 (N=219) | 0.34 (N=513) |
|  | 1 | 0.32 (N=651) | 0.33 (N=227) | 0.33 (N=690) |
|  | 2 | 0.34 (N=211) | 0.38 (N=56) | 0.27 (N=326) |
| **rs942283 (C)** | 0 | 0.31 (N=633) | 0.31 (N=218) | 0.34 (N=518) |
|  | 1 | 0.32 (N=650) | 0.34 (N=230) | 0.33 (N=694) |
|  | 2 | 0.33 (N=213) | 0.37 (N=55) | 0.27 (N=330) |
| **rs942280 (G)** | 0 | 0.31 (N=632) | 0.31 (N=216) | 0.34 (N=512) |
|  | 1 | 0.32 (N=651) | 0.33 (N=227) | 0.34 (N=692) |
|  | 2 | 0.34 (N=213) | 0.37 (N=55) | 0.27 (N=327) |
| **rs942278 (T)** | 0 | 0.31 (N=631) | 0.31 (N=219) | 0.34 (N=513) |
|  | 1 | 0.31 (N=650) | 0.34 (N=227) | 0.34 (N=685) |
|  | 2 | 0.33 (N=212) | 0.38 (N=56) | 0.29 (N=326) |
